# Endogenous insulin secretion in critically ill patients

**DOI:** 10.1186/cc10775

**Published:** 2012-03-20

**Authors:** C Pretty, A Le Compte, J Lin, G Shaw, JG Chase

**Affiliations:** 1 University of Canterbury, Christchurch, New Zealand; 2 Christchurch Hospital, Christchurch, New Zealand

## Introduction

Glucose-insulin system models can be used for improved glycemic control of critically ill patients. A key component of glucose-insulin models is pancreatic insulin secretion. There are limited data in the literature quantifying insulin secretion in critically ill patients at physiologic levels. This study presents a model of pancreatic insulin secretion in critically ill patients based on data from a critically ill population.

## Methods

Samples were collected from 19 patients enrolled in a prospective clinical trial studying sepsis at the Christchurch Hospital ICU. Fifteen of the patients had confirmed sepsis and three were diagnosed type 2 diabetics. All patients were on the SPRINT glycaemic control protocol [1]. Each patient had arterial blood samples assayed for insulin and C-peptide. Two sets of four samples were taken from each patient, with each set collected over 60 minutes. Blood glucose (BG) data were collected with a bedside glucometer. C-peptide data were deconvolved using the model and population parameter values of van Cauter and colleagues [2] to determine pancreatic insulin secretion rates (ISRs). Data from Kjems and colleagues investigating the potentiating effects of glucagon-like peptide-1 on insulin secretion [3] suggested a maximum secretion rate of 16 U/hour. A minimum rate of 1 U/hour was also adopted.

## Results

The best model for insulin secretion was based on blood glucose concentration alone. There was clear separation of secretion levels between normal glucose tolerant (NGT) and impaired glucose tolerant (IGT) patients. Hence, ISR was modeled as a constrained linear function of BG (in mmol/l) for NGT and IGT patients separately with *R*^2 ^= 0.61 and 0.69 respectively. NGT: ISR = 893 × BG - 2,996 (mU/hour). IGT: ISR = 296 × BG - 1,644 (mU/hour). The glucose coefficients of 893 and 296 mU.l/mmol.hour were comparable to data published in a number of other studies for healthy and diabetic subjects.

## Conclusion

This work presents a simple model for pancreatic insulin secretion in critically ill patients based on clinical data. The model is a function of blood glucose level and glucose tolerance status and compares well with published data for healthy and diabetic subjects. This model can be incorporated into glucose-insulin system models and could potentially improve model-based glycaemic control.
